# Inequality persists in a large citizen science programme despite increased participation through ICT innovations

**DOI:** 10.1007/s13280-023-01917-1

**Published:** 2023-09-14

**Authors:** Mari Jönsson, Dick Kasperowski, Stephen James Coulson, Johan Nilsson, Pavel Bína, Christopher Kullenberg, Niclas Hagen, René van der Wal, Jesse Peterson

**Affiliations:** 1 SLU Swedish Species Information Centre, Swedish University of Agricultural Sciences, Uppsala, Sweden; 2 Department of Philosophy, Linguistics and Theory of Science, Gothenburg University, Göteborg, Sweden; 3 The University Centre in Svalbard, Longyearbyen, Norway; 4 Swedish Environmental Protection Agency, Stockholm, Sweden; 5 Department of Ecology, Swedish University of Agricultural Sciences, Uppsala, Sweden; 6 Department of Geography, University College Cork, Cork, Ireland

**Keywords:** Artportalen, Biodiversity data, Biological recording, Citizen science, Inequality, Participant age and gender

## Abstract

Biological recording is a prominent and widely practised form of citizen science, but few studies explore long-term demographic trends in participation and knowledge production. We studied long-term demographic trends of age and gender of participants reporting to a large online citizen science multi-taxon biodiversity platform (www.artportalen.se). Adoption by user communities and continually developing Information and Communications Technologies (ICTs) greatly increased the number of participants reporting data, but profound long-term imbalances in gender contribution across species groups persisted over time. Reporters identifying as male dominated in numbers, spent more days in the field reporting and reported more species on each field day. Moreover, an age imbalance towards older participants amplified over time. As the first long-term study of citizen participation by age and gender, our results show that it is important for citizen science project developers to account for cultural and social developments that might exclude participants, and to engage with underrepresented and younger participants. This could facilitate the breadth of engagement and learning across a larger societal landscape, ensure project longevity and biodiversity data representation (e.g. mitigate gender bias influence on the number of reports of different species groups).

**Supplementary Information:**

The online version contains supplementary material available at 10.1007/s13280-023-01917-1.

## Introduction

Citizen science (e.g. sometimes also named community, crowd, civic, or participatory science) focusses on the involvement of volunteer participants in processes of data and scientific knowledge production, although it remains a flexible concept that is hard to define (e.g. Kullenberg and Kasperowski 2016). Biological recording is one of the oldest, largest and most established fields of citizen science globally (Silvertown 2009). Through this, participants have the opportunity to learn about research and its processes, to contribute directly to research projects with their intellectual skills and expertise and to make an impact on science and society (Bonney et al. 2014). Resulting species records are often rapidly accumulated in regional or national data infrastructures (e.g. Swedish Species Observation System, or ‘Artportalen’,^1^ and eBird) and then further aggregated on a global scale, such as through the Global Biodiversity Information Facility (GBIF^2^). Such ‘Big Data’ is often denoted by huge amounts of observations, reported in near real time, diverse in structural variety and quality (e.g. resolution, content, reusability and scalability), and large in spatio-temporal scope (Kitchin 2014; Kelling et al. 2015). For environmental citizen science data, such as biological records by volunteers, to be trusted and broadly applied in research and society the observations need to be of high or at the very least known quality, timely and accessible (Theobald et al. 2015; Fritz et al. 2019). This requires supportive and highly developed Information and Communications Technologies (ICTs) (Pocock et al. 2018). Indeed, the rapid increase in accessible digital technologies and data-science tools (e.g. computers, smartphones, mobile platforms, wireless networks and databases) continue to transform citizen engagement and knowledge production in environmental science and resource management (Arts et al. 2015), now also recognized by the UN as necessary for monitoring Sustainable Development Goals (Fraisl et al. 2020). Continued high inclusion of members of the public, is crucial to the successful development and application of long-term citizen science projects, such as biological recording platforms crucial in societal decision making (Kasperowski and Hagen 2022).

The word ‘citizen’ implies a relationship to a State or commonwealth, but inherently also inclusion of diversity in terms of age, gender, ethnicity, geography and social class. Findings from studies on gender composition among participants in citizen science are mixed; some projects, however, show a strong bias of participants identifying as male (Paleco et al. 2021). Large citizen science platforms such as eBird at the Cornell Lab of Ornithology, display that the majority of participants are highly educated, upper-middle class, middle-aged or older and white (Purcell et al. 2012). It has been argued that there is a moral and ethical responsibility to include a more diverse group of participants, to bring a broader range of perspectives to inform citizen science projects and provide access to their benefits (Mor Barak 2018). Benefits include participants’ knowledge of the subject (Brossard et al. 2005; Jordan et al. 2011), increased science literacy (Crall et al. 2013; Bonney et al. 2014), engagement in conservation activities (McKinley et al. 2017; Lewandowski and Oberhauser 2017), increased environmental advocacy and networking (Johnson et al. 2014) and more positive attitudes towards nature (Sharma et al. 2019). Participants might even develop knowledge beyond the tasks that scientists originally mobilized them to perform, even creating interests interfering with those tasks, but also contribute to scientific discoveries (Cornwell and Campbell 2011; Kasperowski and Hillman 2018). It is, therefore, important for citizen science project developers and end-users to understand the long-term trends in the demographics of volunteers participating in citizen science. In addition, trends in long-term level of engagement (e.g. contributed tasks, time invested) are also important to consider in relation to the demographics of participants. Citizen science projects are often characterized by a smaller proportion of highly motivated long-term volunteers contributing extensively with knowledge and time (Silvertown et al. 2015; Seymour and Haklay 2017). Projects that rely on exclusive communities risk losing highly specialized, skilled and committed participants, if implemented project tools (e.g. relevant ICTs) and benefits fail to match the needs and skills of these volunteers. The level of engagement and development across age and gender of participating volunteers in relation to the long-term development of relevant ICTs have received limited scholarly attention (Lemmens et al. 2021). Potentially, engagement and contributed data and knowledge may change over time as a result of changes in participant age and gender structure, but this type of data is not regularly collected in citizen science projects (c.f. Moczek et al. 2021). For example, retired citizens often contribute substantially with both time and knowledge to citizen science projects (Land-Zandstra et al. 2021). At the same time, the recruitment and engagement of younger participants over time is clearly essential to the continuity and success of large-scale and long-term projects. Age and gender-related changes in participation may occur, not only as a result of ICT access and developments, but also as a result of cultural and social developments in projects as well as general societal characteristics in population age structure, gender equality, geography, socio-economic factors and interests in science (Mac Domhnaill et al. 2020). To what extent the participants in citizen science ought to reflect the demographics of the wider population remains an ambiguous question (Spiers et al. 2019). Some projects benefit highly from being exclusive, mobilizing expertise knowledge beyond the realm of professional scientists (Koepnick et al. 2019). Should citizen science project managers aim to balance participation across age and gender, dependent or independent of the wider population and in relation to the goals of the specific project? Does the age and gender structure matter for the personal benefits of participating volunteers, e.g. in terms of motivations, learning and interactions with peers and within networks? Or in terms of overall project outcomes, e.g. amount and quality of data and knowledge contributed? To start answering these questions, we need baseline information of the long-term trends in age and gender participation in citizen science projects that can be related to data and knowledge production, benefits for volunteers, ICT developments and other societal changes occurring over time. Knowledge of such long-term trends in age and gender participation in online citizen science are currently lacking. Here we primarily focus on how age and gender trends in participation may influence the amount and type of biological data recorded.

Several countries have a long legacy of biological recording, developed through the skills and commitment of volunteer participants (Sutherland et al. 2015). However, few online large-scale standardized projects do trace both biological recordings and citizen participation over longer time spans. The Swedish Species Observation System, or Artportalen in Swedish, is the largest national multi-taxon biological recording citizen science online platform contributing to GBIF. Artportalen was initially composed of taxa-specific portals, which have over time been merged into one more advanced multi-taxon system with a greater breadth of digital technologies and data-science functionalities. The ICT development of the system represent a ‘good study case’ of a large-scale and long-term biological recording citizen science platform. As the largest multi-taxon data provider to GBIF, the citizen-generated Artportalen data are used in hundreds of international scientific papers and applications (949 citations in Dec 2022;^3^). The Artportalen data are used extensively in societal decision making and knowledge production for environmental governance nationally in Sweden (e.g. Kasperowski and Hagen 2022) and internationally (e.g. EU Species and Habitat Directives, international Red Lists, global management of invasive species). The Artportalen system also form the backbone for building similar systems in other Nordic countries. The participants (active reporters) are visible in the Artportalen system through personal accounts that are linked to over > 99 million geo-referenced observations (Aug 2023) across multiple species groups. This offers unique opportunities to study the trends in participation across age and gender for a range of taxa over almost two decades, providing novel knowledge that is of high international relevance. We hypothesize that participation is biased towards middle-aged or older men based on current knowledge (Purcell et al. 2012; Wright et al. 2015; Mac Domhnaill et al. 2020; Paleco et al. 2021), but that such a bias or ‘age and gender gap’ converge (i.e. become smaller) over time. We have limited scientific support for this second part of the hypothesis concerning the long-term temporal trends, but base this on general societal improvements in access to ICTs and an overall aim of citizen science to be accessible to all people (ECSA 2015). We analyze how age and gender may influence the amount and type (i.e. species groups, species list lengths) of biological data recorded and relate our findings to Artportalen ICT developments and the scientific literature on social and cultural aspects of citizen science projects. The resulting long-term trends provide important baseline information for citizen science project facilitators and future research of the development and application of large-scale multi-taxon citizen science platforms.

## Materials and methods

### Artportalen

Artportalen is a national open data platform for reporting and storing species occurrence records from multiple species groups that has been developing over approximately two decades (Table 1, modified after Knape et al. 2022). Artportalen has attracted over 13,000 unique participants reporting geo-referenced observations in 2021, often together with detailed observation information, images, video or sound files. Between 5 and 6 million species occurrences are currently reported each year, the majority presence-only records from volunteers. Artportalen’s societal use—as species data and knowledge base–aligns with interests of researchers, decision makers and wider members of the public (including participants themselves). Government agencies at local and regional authorities in Sweden use data from Artportalen on a daily basis for decisions on environmental issues in the use of land and water (Kasperowski and Hagen 2022). Their study also revealed that trust in Artportalen data is not uniform but concentrated on observations gathered by a core set of reporters. In Artportalen the reporter is required to remain visible in the system with a login and contact details. Although data on age and gender are not obligatory, the vast majority of Artportalen reporters choose to register their age and gender as man, woman or do not want to reveal. We assigned age and gender (man or woman) to a minimum of 98% of reporters across the species groups studied.

**Table 1:** Major changes to the citizen science reporting system and functionality over time, modified after Knape et al. (2022)

Year		Description of system change
2000	June	Launch of the web platform for birds as ‘Artportalen’
2003	Autumn	Launch of the web platform for vascular plants
2003	Autumn	Launch of the web platform for butterflies and moths
2003	Winter	Launch of the web platform for fungi
2006	Spring	New web platform for birds (replacing the former platform). First reports of bryophytes (i.e. earlier bryophyte records were reported from past data records)
2006	Autumn	New web platform for invertebrates (including insects and spiders)
2007	Spring	Launch of the web platforms for non-bird vertebrates and fish
2007	Summer	New multi-taxon web platform for non-animal groups (algae, bryophytes, fungi, lichens and plants)
2007	Autumn	Launch of the web platform for marine invertebrates
2013	May	New multi-taxon web platform ‘Artportalen 2’ to merge several of the former separate platforms: all non-animal groups merged with all non-bird vertebrates
2014	May	Inclusion of the web platform for invertebrates from 2006 in the multi-taxon Artportalen 2
2015	April	Inclusion of the web platform for birds in the multi-taxon Artportalen 2. Artportalen 2 is now complete with all species groups merged
2019	Spring	Launch of new functionalities and possibility to report via both mobile phone devices and desktop. Also implementation of checklist app for birds, with easier in-field reporting and reporting of species absences

### Data extraction

For seven different species groups (birds, bryophytes, fungi, invertebrates, lichens, non-bird vertebrates and vascular plants) and gender (men and women), we extracted yearly data from 2003 to 2020 on (i) number of reporters, (ii) number of unique species reported, (iii) number of field days spent reporting, (iv) mean number of species reported per field day (hereafter mean species list length) and (v) reporter age. For (i) the number of reporters and (ii) number of unique species reported we used the record registration date to assign year. We define a reporter as a registered Artportalen user that actively contribute with species data at least once. We analyse full reporter communities each year, allowing turnover of reporters over time. We do not follow the same ‘cohort’ of reporters over time; even if long-term reporters are part of communities and their important contribution are visible via reporter ranking lists. For (iii) number of field days spent reporting and (iv) mean species list length we used the observation date to assign year. For species reported and reporting activities, species occurrences (presence-only data) make up the vast majority of records contributed and were, therefore, analysed in this study. Hence, the pseudo-absence data generated from the implementation species checklists for birds from year 2019 onward (Table 1) were not included as species reports or reporting activities in this study, since they make up a recent special case for birds only. The vast majority of records refer to terrestrial ecosystems. However, three of the species groups (non-bird vertebrates, invertebrates and vascular plants), also include good numbers of records from aquatic ecosystems.

### Analyses

We modelled long-term trends in the numbers of (i) reporters, (ii) species reported, (iii) field days reporting and (iv) young reporters (< 30 years old) over time (years) and by gender using generalized additive models (GAMs) with a log(ln) link and a negative binomial distribution. We modelled the long-term trend using a thin plate spline with 10 degrees of freedom. To model (v) the number of species reported yearly over time (year and year^2^, assuming there could be an intermediate optimum) and gender, we used linear models on log(ln) transformed response data. To model (vi) mean species list length and (vii) mean age, over time (year and year^2^) and gender, we used generalized linear models with Poisson distribution. We visualized the age-class distribution as the proportion of reporters over time in bar charts.

We conducted all statistical analyses in R version 4.03 (R Core Team 2020). GAM models were fitted using the functions gam in the R package mgcv (Wood 2006) and figures were produced using the package ggplot2 (Wickham 2016).

## Results

### Trends in reporter numbers

**Fig. 1:**
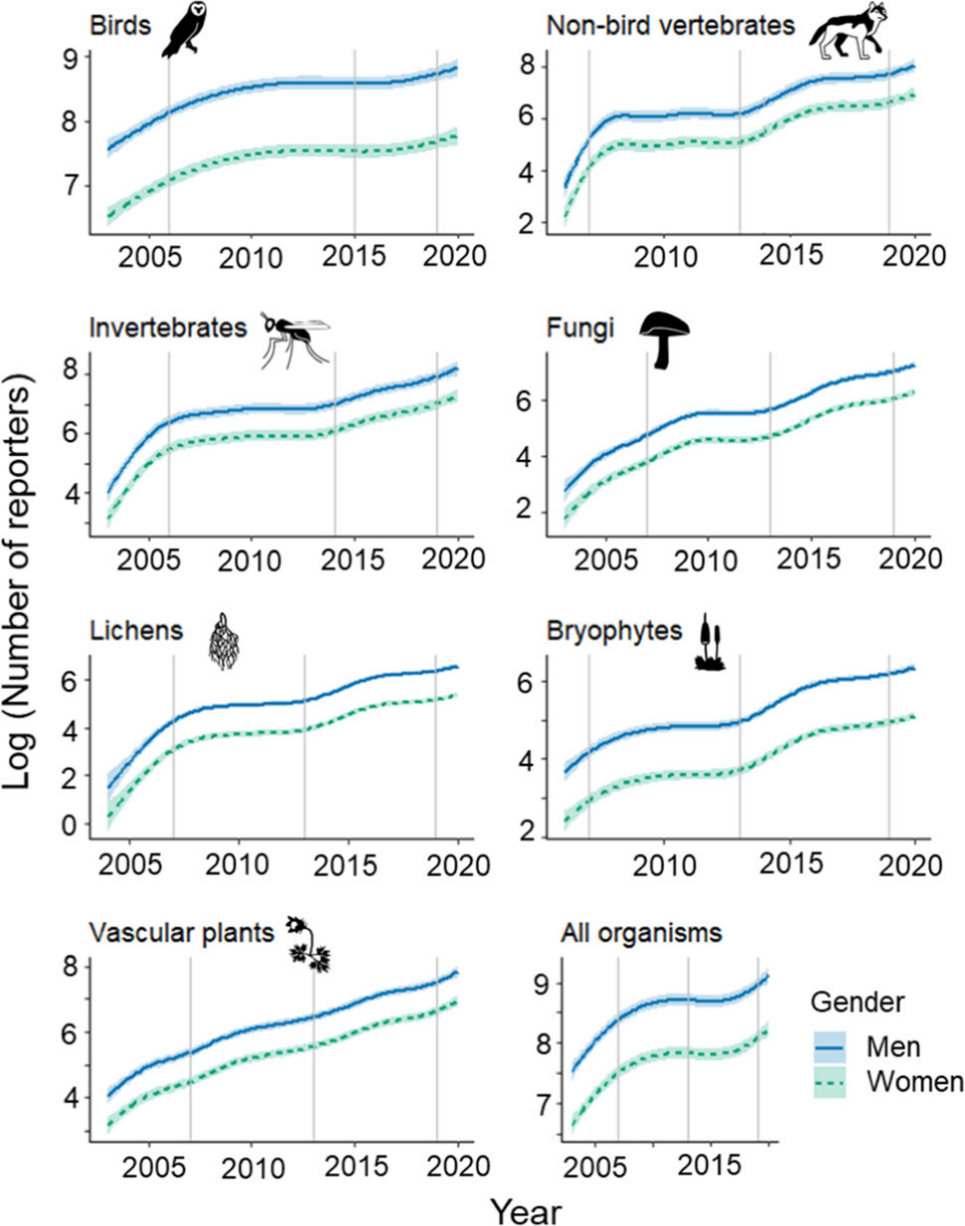
Estimated yearly logarithmic number of men and women reporting in different species groups. Trends (with 95% confidence intervals) are predicted values estimated from the generalized additive models. Vertical lines indicate times when new ICT developments were introduced (see Table 1). Note that the scales of the *y*-axes differ

The number of reporters engaged generally increased over time, although the temporal trend in numbers varied with the species group and gender (trends on natural logarithmic scale, Fig. 1). In the supplementary material we include plots of raw data points and predicted trends on the original scale (Fig. S1). Right from the start, there was a clear bias towards individuals identifying as men in the numbers of reporters to Artportalen across species groups, and this gender gap remained constant over time. Increasing numbers of active reporters of both men and women often followed the introduction of new ICT applications, but the magnitude and extent of such increases varied among species groups.

### Trends in biodiversity reported

**Fig. 2:**
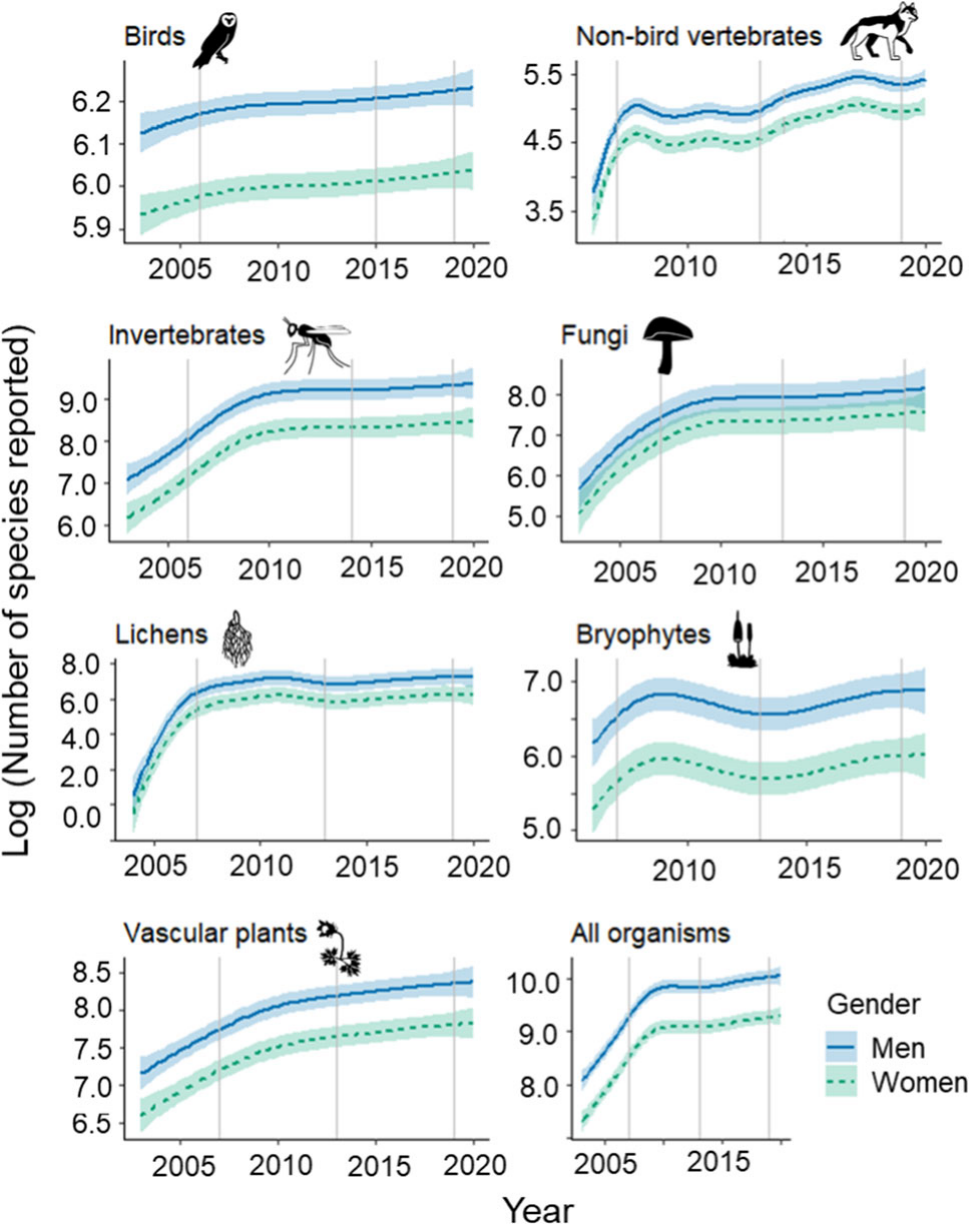
Estimated yearly logarithmic number of species reported by men and women. Trends (with 95% confidence intervals) are predicted values estimated from the generalized additive models. Vertical lines indicate times when new ICT developments were introduced (Table 1). Note that the scales of the y-axes differ

The temporal trend in the number of unique species reported increased year upon year, though for several species groups an asymptote was reached or approached after around year 2010 (Fig. 2, Supplementary material Fig. S2). The introduction of new ICT applications had a positive impact on the number of species reported in some species groups. Men generally contributed more towards the biodiversity reported than women, with differences being smallest for fungi and lichens (see confidence intervals). Considering all organisms, men reported on average twice as many species (> 20 000 species per year) as women (around 10 000 species per year) across all species groups over the last decade (raw data in Fig. S2). This is perhaps not surprising given the greater number of men contributing with data, but it could also be related to differences in levels of engagement and skills, e.g. men finding new and rarely reported species from having greater access to species-expert networks and being more motivated by increasing levels of competition and species ranking lists (NASEM 2018).

### Trends in reporter field days

**Fig. 3:**
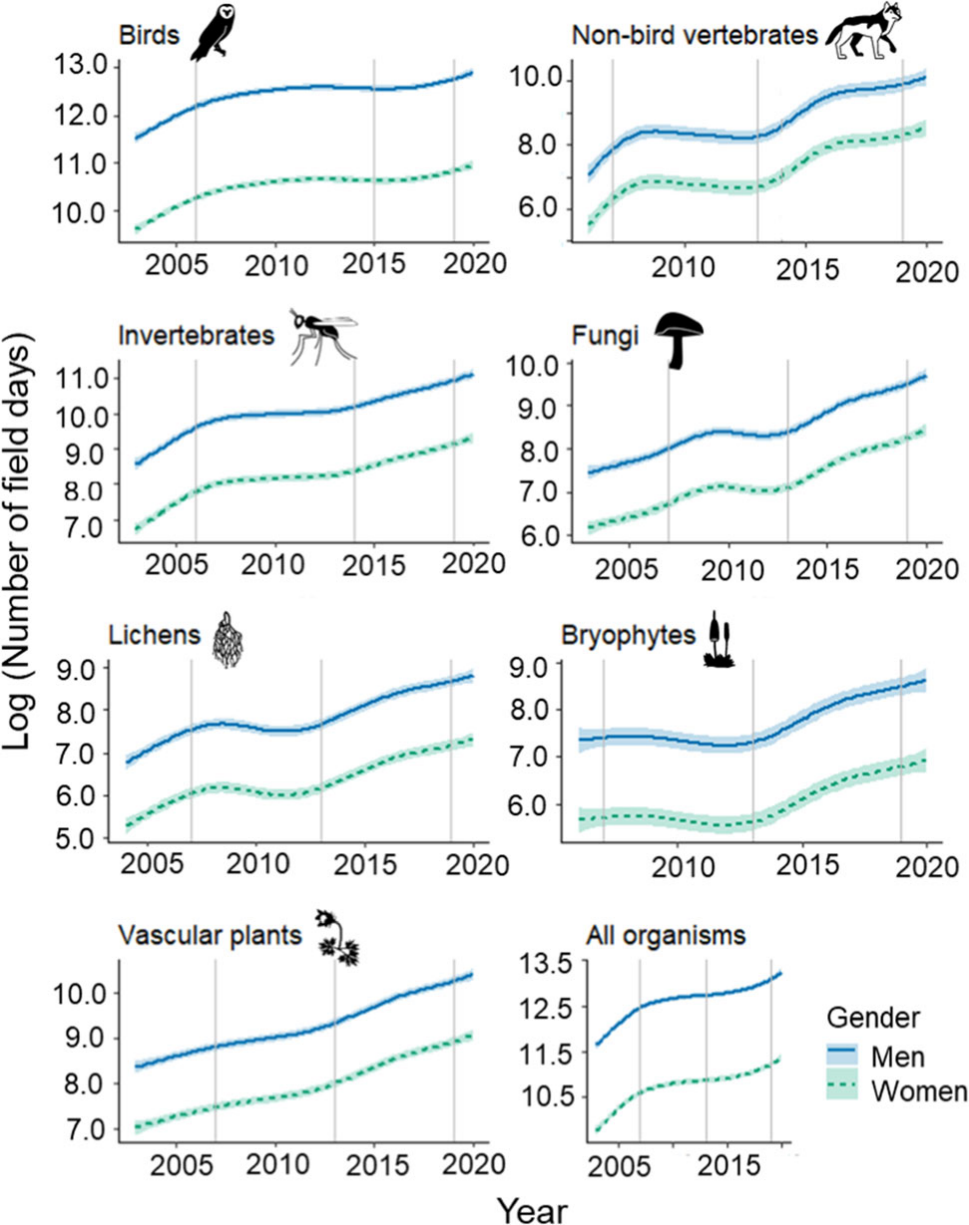
Estimated yearly logarithmic number of days spent in the field by men and women. Trends (with 95% confidence intervals) are predicted values estimated from the generalized additive models. Vertical lines indicate times when new ICT platforms were introduced (Table 1). Note that the scales of the y-axes differ

The number of field days spent reporting increased over time, although the temporal trend in numbers varied with species group and gender (Fig. 3, Supplementary material Fig. S3). There was a substantial bias towards men in the number of field days spent reporting to Artportalen across all species groups. The introduction of new ICT applications, especially the multi-taxon web developments in 2013–2015, followed by increased reporting field days but without lessening the gap between men and women.

### Trends in mean species list length

**Fig. 4:**
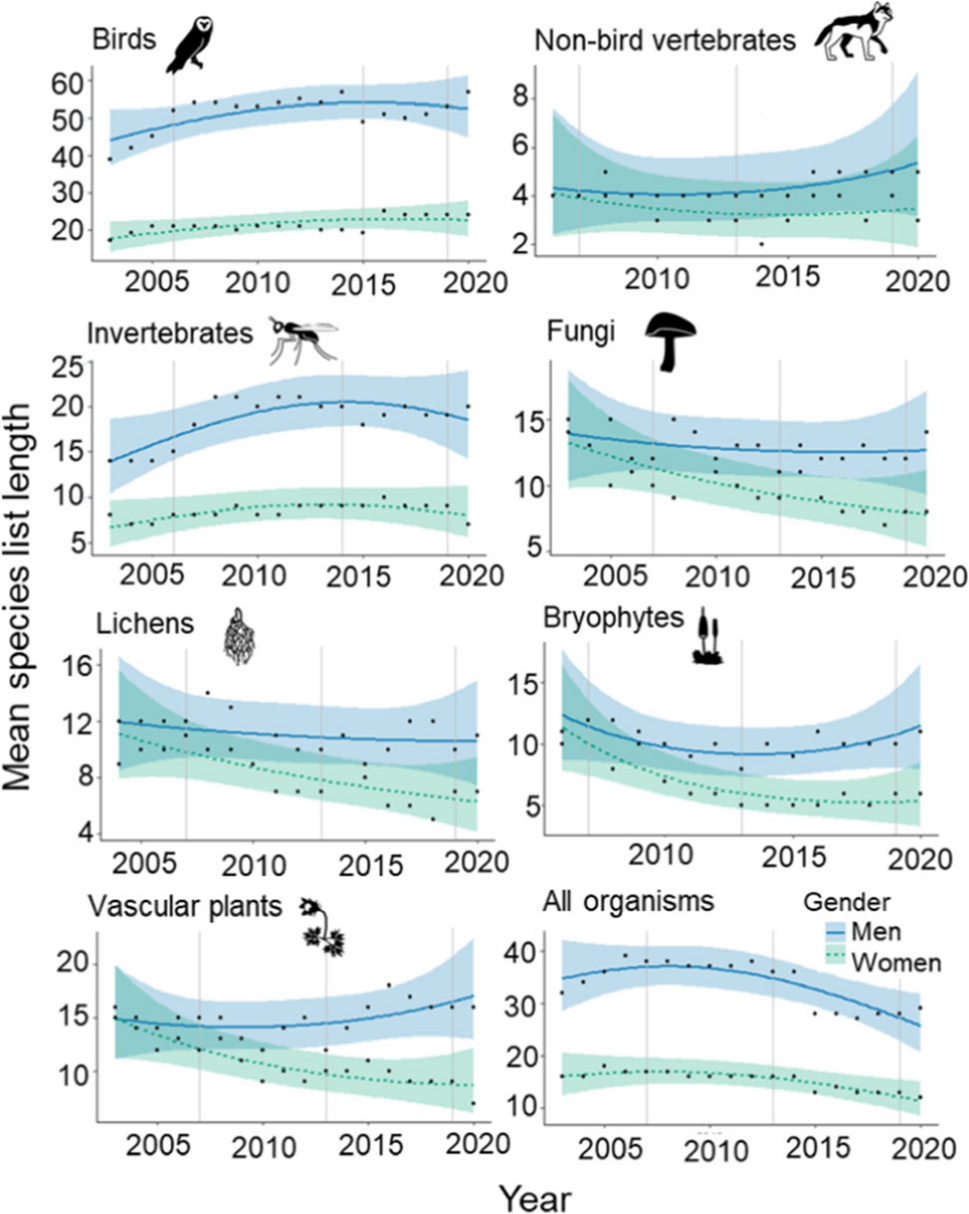
Estimated yearly mean species list length reported per field day by men and women (black dots represent raw data). Trends (with 95% confidence intervals) are predicted values estimated from the generalized linear models. Vertical lines indicate times when new ICT developments were introduced (Table 1). Note that the scales of the *y*-axes differ

Men on average recorded many more species per field day compared to women, especially for birds, invertebrates and over time also for bryophytes and vascular plants (Fig. 4). The introduction of new ICT applications did not seem to consistently impact long-term trends in mean species list length, although somewhat fewer birds and insects were initially reported by men with the introduction of new ICT applications in 2013–2015. The 2019 ICT innovation (mobile reporting) coincided with a reduction in list length for women in three groups (invertebrates, vascular plants, non-bird vertebrates), while for men it coincided with an increase in three groups (lichens, fungi, bryophytes).

### Reporter age trends

**Fig. 5:**
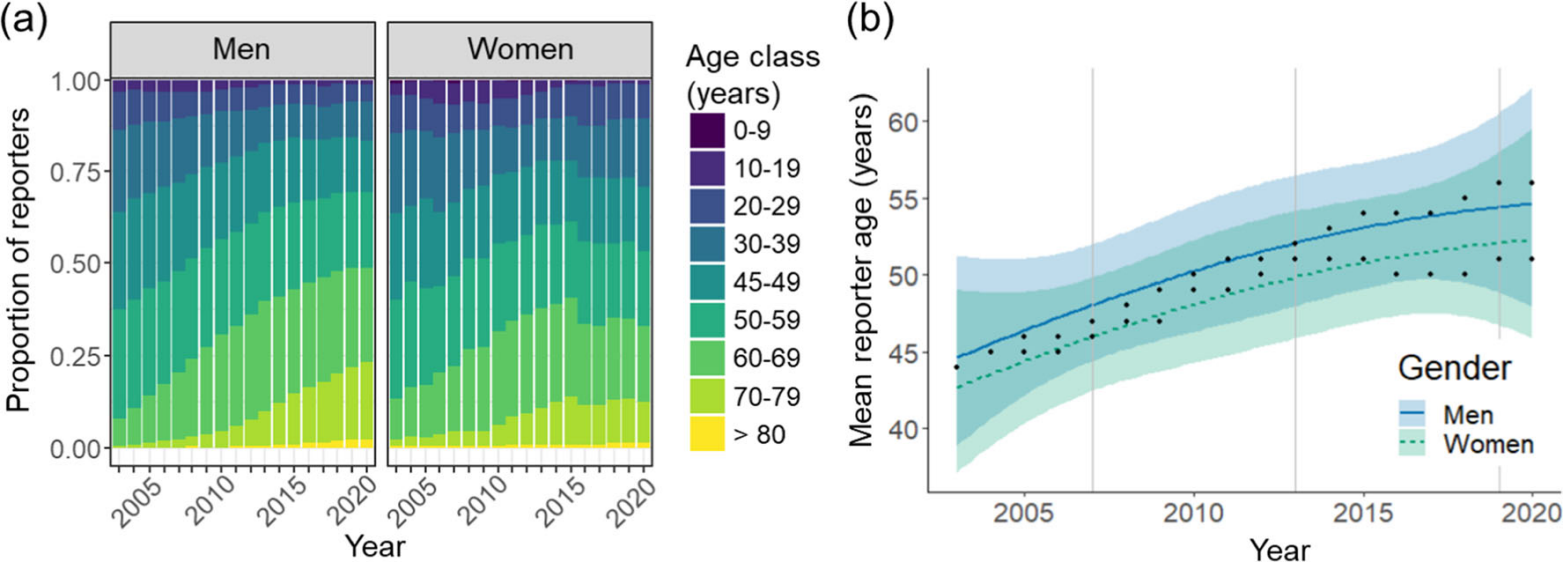
Age-class distribution (**a**) and estimated mean age (**b**) of men and women reporting all organisms to Artportalen over time. Trends for mean reporter age (with 95% confidence intervals) are predicted values estimated from the generalized linear models (black dots represent raw data). Vertical lines indicate times when new ICT applications were introduced in year 2007, 2013 and 2019

The age-class distribution skewed towards dominance of older reporters over time, and this was more pronounced for men than women (Fig. 5a, Supplementary material Fig. S4). The number of participating young men and women (< 30 years old) increased after the introduction of new ICT applications 2013–2015, but generally more so for men than women (Supplementary material Fig. S5). Still, changes in the age distribution resulted in increasing mean reporter age for both men and women over time (Fig. 5b) and across species groups (Supplementary material Fig. S6). The age-class distribution showed that invertebrate reporters were generally somewhat younger than reporters of other species groups (Supplementary material Fig. S4). The mean reporter age increased from 44 years in 2003 to 55 years in 2020, when considering both genders and all species groups. Gender differences among young reporters (< 30 years old) tended to be smaller for several species’ groups (non-bird vertebrates, fungi, bryophytes) through time (Supplementary material Fig. S5), pointing to higher retention of women.

## Discussion

Our results highlight imbalanced participation in respect to both age and gender. Regarding gender, our results agree with previous research on the gender imbalances in online citizen science projects, which tend towards male participants (Wright et al. 2015; Mac Domhnaill et al. 2020; Paleco et al. 2021). The gender gap remained remarkably constant over time, although the magnitude of the gender gap varied substantially across species groups. This does not agree with our temporal trend hypothesis that the gender gap would converge over time, and the gender gap in estimated yearly mean species list length reported per field day actually increased over time for several species groups. The gender imbalance in Artportalen (about 32% female in 2020) mirrors other collaborative online citizen science projects, such as Zooniverse (about 30% female contributors across 17 different projects; Ibrahim et al. 2021), as well as gender distribution in more formal scientific activities. For example, despite policy changes and targeted outreach in the natural sciences, issues of underrepresentation persist for women and minorities in many fields (Huang et al. 2020).

The reasons for such imbalances may be explained by underlying historical, social and cultural factors. Artportalen started as a project about birds by birders (Aronsson et al. 2013), and birding typically has been an activity that skews towards white males in their middle-age or older (Cashman-Brown 2012; Wright et al. 2015; Edwards et al. 2018). Coming from a male-dominated culture, it is likely that Artportalen reinscribed many of the same values (e.g. individual and social learning opportunities, data storage and visualization, ranking lists) in its reporting system as pre-internet bird reporting culture. For instance, Artportalen’s digital interface and architecture—such as species lists and rankings, the handling of rare findings and how data are structured for the end-user (e.g. reports per year, number of unique species)—reproduce values concerned around competing, observing and reporting in large quantities and on the rare and exceptional. That is, the value of any given participant, as evidenced by reporter ranking lists as well as what species get reported, increases based upon the number of records they produce as well as how adept they are at reporting rarities (notified in local and national communities via apps and social media). In this sense, the culture of Artportalen rewards rarity and individuation (i.e. personal identity related to effort and skills). The longer an individual remains active on Artportalen and produces rare records, the more recognizable and trusted they become to the system’s managers and users. Thus, participants undergo a socialization process that co-produces inequality and separation from the ‘usual’ both in terms of what one reports, and the way members of this culture are valued. In other words, Artportalen produces practices (epistemic and cultural) that afford the special competitiveness and social obligation present in birding, which in turn may have originated from a very male-dominated amateur culture (Lundquist 2018). This also agrees with research showing that long-term participants are often motivated by social obligation, a shared ideology, helping others and a feeling of satisfaction, while new participants are often motivated by egocentric gains or curiosity about a project (Land-Zandstra et al. 2021). Artportalen data are largely contributed by private individuals, but some data are also reported by professionals in various fields, such as forestry, conservation, hunting, farming, fishing and other outdoor activities. Gender and age biases in Artportalen submissions, could, therefore, be somewhat influenced by such professional activities. Yet, we have no reason to believe that the reported gender and age biases observed are due to contributions of professionals.

Having come from this specific context, it is reasonable to assume that such a system reproduces it in the digital for its members, even if one aim is to increase participation as much as possible. Previous research on the behaviour of volunteer participants in citizen science shows how motivations encourage participation by some over others (Phillips et al. 2019). Men and younger individuals have been found to be more influenced by extrinsic motives (external cultural benefits or rewards, recognition of effort), while women and older persons are driven more by intrinsic motives (Lakomý et al. 2020; Larson et al. 2020). Our results indicate that online citizen science biodiversity platforms such Artportalen must accommodate for extrinsic and intrinsic motivations (e.g. individual and social learning opportunities, data storage and visualization, contribution to science and conservation), to encourage broad participation. On the other hand, the results also suggest that Artportalen has been very successful in terms of longevity, overall biodiversity data and knowledge production (Peterson et al. 2022, 2023a). As such, and given limited resources for development, we argue that platforms such as Artportalen often do not prioritize accommodating additional motivations or benefits because *it does not need to* from a utilitarian scientific context of ‘reporting effort and data capture over time’. However, it is important to acknowledge that biased inclusiveness could affect data quality and have wider implications for data collection and societal usage (ECSA 2015), but we did not study this. For example, data quality can be reduced if participants do not cover certain spatio-temporal spaces because they are spaces with predominantly women and younger participants. In addition, citizen science involving marginalized and/or indigenous communities could have a significant influence on participants thereof (ECSA 2015; Benyei et al. 2023). However, it must not be normatively induced that this is positive or of benefit for individuals, groups or society at large. How citizen science affect society is an empirical question. We suggest that future research should investigate how gender inclusiveness and engagement can (i) introduce biases in species identification and distribution across time and space, as well as influence the types of environmental metadata collected, and (ii) contribute to a more comprehensive definition of success, such as enhancing co-creation of knowledge, skills, networks and stakeholder participation (Pandya 2012; Gardiner and Roy 2022) and (iii) shape human-nature relationships including the potential development of environmental citizenship (Benyei et al. 2023; Peterson et al. 2023b).

In respect to age, our results compare with earlier research showing age imbalance toward older participants in online citizen science projects (e.g. Purcell et al. 2012; Wright et al. 2015; Mac Domhnaill et al. 2020; Cooper et al. 2021). We also reveal clear ageing trends among both male and female participants, without this converging the age and gender gap. We also show that the ageing profile of participants occurred across multiple species groups and was often more pronounced among men. For several species groups, the mean age of reporters increased by approximately eleven years over almost two decades. This finding could mean that a higher number of older participants create Artportalen accounts and report species compared to younger participants, that the pace of registration and reporting among younger participants has slowed (especially for birds), or both. The successional ageing of registered and active long-term/systematic reporters could also influence these trends. Thus, even with Artportalen-specific and general societal improvements in access to ICTs and good practices in citizen science (ECSA 2015), young people (here also including young adults in their 20 s) participate less in citizen-based biological recording.

### Technological considerations

In respect to the recording platform itself, we show that Artportalen’s ICT developments do not necessarily overcome its existing gender and age imbalances. Our data strongly suggest that technological development, in terms of the addition of better interfaces, mobile applications and data computation tools, does not change social structure; rather the opposite, it seems to have no effect on social imbalances over time. This finding is supported by historical, social and gender studies of technology that show how gender is expressed through technologies (Cockburn and Ormrod 1993; Mackenzie and Wajcman 1999; Baym 2015) and that technical skills and domains of expertise shape masculinities and femininities (Bray 2007). Studies on ageism and technology also demonstrate how digital technologies produce stereotypes portraying older adults as incapable and technophobic, which influence ICT development and ultimately the participation of different age groups (Mannheim et al. 2019; Köttl et al. 2021). From initial design to implementation, ICTs often perpetuate exclusionary values and, based on our study, appear to remain entrenched because updates do not address the underlying assumptions built-in to the existing infrastructure and because the cultural momentum of users and their values also demand innovation for the existing structure to continue to serve their ends and not others.

Thus, although continuously developing and implementing ICTs is essential to citizen-generated online biological recording, as with Artportalen, such updates may not affect overall imbalances in participation without overhauling the entire system and the values inscribed in its infrastructure. Although the development and introduction of new ICTs in Artportalen were followed by a short time increase in numbers of reporters and contributed data, they generally did not reduce age and gender imbalances among participants nor increase the number of species reported per field day visit. Imbalances towards older male participants instead generally amplified over time. Hence, Artportalen ICT development can be understood to primarily cater for the user base community and perpetuate the existing social structure and epistemic culture established prior to its release in 2000.

Further research on demographics in long-term citizen science projects is needed, especially to understand the impacts that technology might play in shaping who uses them. Longer time series than approximately two decades may be needed to improve our understanding of the role of participation for trends in biodiversity and ICT improvements that sometimes occurs over longer time scales. The development of technologies that employ novel biological detection and identification methods (e.g. automated monitoring, computer vision, molecular methods and radar; van Klink et al. 2022) can potentially influence future participation and biological recording in citizen science programmes. As we present in this article, more research on imbalances in age and gender (and other factors not covered such as class, race and ethnicity) need to be understood over time, especially in projects that have been ongoing for decades. Moreover, research needs to address the relative benefits and costs of creating more and more applications that might cater to specific, exclusive communities, such as iNaturalist’s new (image recognition) application “Seek”^4^ and Artportalen’s gamified environment biologg,^5^ which target younger users and families. Promoting higher levels of engagement, such as through collaborative or co-created projects where engagement in the scientific process goes beyond data gathering (i.e. formulating relevant questions, adding and interpreting data on own initiative and disseminating results), have been proposed to overcome some historical barriers to inclusion (Pandya 2012; Gardiner and Roy 2022). Artportalen successively implement tools to empower reporters to co-creatively provide their ecological knowledge of the location and the species, i.e. the possibility to design and manage projects with own parameters, create field visits to write diary entries and document circumstances and adding various media files. The influence of such developments on participation and data representation are yet to be documented. Finally, we lack long-term information on a number of other factors that may be temporally dynamic and influence participation over the course of a long-term citizen science programme, e.g. individual and community-level motivations, education, socio-economic status, language and access to natural areas (Pateman et al. 2021; Lewenstein 2022). In fact, motivational and demographic factors are rarely documented and published over the course of a citizen science programme, but important to monitor diversity in citizen science (Pateman et al. 2021).

## Conclusions

Imbalances in citizen science are important to bring forward and further explore, as citizen science is currently undergoing a process of institutionalization, which evokes positive visions and high expectations of democratization, trust and scientific literacy as societal benefits. Yet, imbalances in age and gender over 20 years in one of the world’s largest multi-taxa biodiversity databases—enduring even through ICT innovations and updates—point to serious challenges for practising more inclusive science. Specifically, three key issues emerge.

First, the contexts from which many digital citizen science projects emanate—having been institutionalized for decades—may prevent more inclusive and equitable participation. Artportalen, for instance, emerged from a male-dominated birding culture that reproduces itself in the digital. That is, social aspects within birding carry over into biological recording, such as lists and interests in rare sightings. Our research shows that it is important for citizen science project facilitators to engage more broadly with women and younger participants in order to maximize the breadth of engagement and learning across the larger societal landscape. Hence, managers and operators of such projects should seek frameworks and methods for increased awareness and collaboration from a more inclusive public.

Second, ICT developments, updates and releases typically reinforce the assumptions and values already implicit within the infrastructure. Clearly, the development of ICT systems for citizen science would benefit from developments currently lacking in the literature, as studies largely remain concerned with motivation. The age of projects and infrastructures further represents a significant challenge for ushering citizen science into a more equal representation of participants. To change such infrastructures, their operators would need much more financial, institutional and social science support to address issues of inclusion and equity.

Third, normative thinking about biological records would insist on the need for ever more data at greater spatial and temporal scales to achieve knowledge about global biodiversity. Thus, success in citizen science is often determined by increasing data capture over time. Such a benchmark does not address values such as inclusivity, diversity or citizenship. In this respect, age, gender, class, ethnicity and more do not matter. Hence, it is not surprising that Moczek et al. (2021) find that citizen science projects in Germany know little about their contributors. In this case, the saying ‘ignorance is bliss’ holds true as exclusive communities produce large amounts of data and, thus, make this citizen science model successful. Yet the cultures of citizen science and biodiversity databanks may have to engage with underrepresented and younger participants, to facilitate the breadth of engagement and learning across a larger societal landscape, ensure project longevity and biodiversity data representation.

### Supplementary Information

Below is the link to the electronic supplementary material.

Supplementary file1 (PDF 1433 KB)

## Footnotes

1 www.artportalen.se

2 www.gbif.org.

3 www.gbif.org/dataset/search.

4 https://www.inaturalist.org/pages/seek_app.

5 https://www.biologg.se/.

## References

[CR1] Aronsson M, Nilsson J, Tano Graflind A (2013). Nya Artportalen. [New Artportalen]. Fauna och Flora: en populärbiologisk tidskrift.

[CR2] Arts K, van der Wal R, Adams WM (2015). Digital technology and the conservation of nature. Ambio.

[CR3] Baym NK (2015). Personal connections in the digital age.

[CR4] Benyei P, Skarlatidou A, Argyriou D, Hall R, Theilade I, Turreira-García N, Latreche D, Albert A (2023). Challenges, strategies, and impacts of doing citizen science with marginalised and indigenous communities: Reflections from project coordinators. Citizen Science: Theory and Practice.

[CR5] Bonney R, Shirk JL, Phillips TB, Wiggins A, Ballard HL, Miller-Rushing AJ, Parrish JK (2014). Next steps for citizen science. Science.

[CR6] Bray F (2007). Gender and technology. Annual Review of Anthropology.

[CR7] Brossard D, Lewenstein B, Bonney R (2005). Scientific knowledge and attitude change: The impact of a citizen science project. International Journal of Science Education.

[CR8] Cashman-Brown O, Falkof N, Cashman-Brown O (2012). Birds of a feather: The whiteness of birding. On whiteness.

[CR9] Cockburn C, Ormrod S (1993). Gender and technology in the making.

[CR10] Cooper CB, Hawn CL, Larson LR, Parrish JK, Bowser G, Cavalier D, Dunn RR, Haklay M (2021). Inclusion in citizen science: The conundrum of rebranding. Science.

[CR11] Cornwell ML, Campbell LM (2011). Co-producing conservation and knowledge: Citizen-based sea turtle monitoring in North Carolina, USA. Social Studies of Science.

[CR12] Crall AW, Jordan R, Holfelder K, Newman GJ, Graham J, Waller DM (2013). The impacts of an invasive species citizen science training program on participant attitudes, behavior, and science literacy. Public Understanding of Science.

[CR13] ECSA (European Citizen Science Association) (2015). Ten principles of citizen science.

[CR14] Edwards R, Kirn S, Hillman T, Kloetzer L, Mathieson K, McDonnell D, Phillips T (2018). Learning and developing science capital through citizen science.

[CR15] Fraisl D, Campbell J, See L, Wehn U, Wardlaw J, Gold M, Moorthy I, Arias R (2020). Mapping citizen science contributions to the UN sustainable development goals. Sustainability Science.

[CR16] Fritz S, See L, Carlson T, Haklay M, Oliver JL, Fraisl D, Mondardini R, Brocklehurst M (2019). Citizen science and the United Nations sustainable development goals. Nature Sustainability.

[CR17] Gardiner, M. M., and H. E. Roy. 2022. The role of community science in entomology. *Annual Review of Entomology* 67: 437–456.10.1146/annurev-ento-072121-075258. 10.1146/annurev-ento-072121-075258 34644156

[CR18] Huang J, Gates AJ, Sinatra R, Barabási AL (2020). Historical comparison of gender inequality in scientific careers across countries and disciplines. PNAS.

[CR19] Ibrahim K, Khodursky S, Yasseri T (2021). Gender Imbalance and spatiotemporal patterns of contributions to citizen science projects: The case of zooniverse. Frontiers in Physics.

[CR20] Johnson MF, Hannah C, Acton L, Popovicia R, Karantha KK, Weinthala E (2014). Network environmentalism: Citizen scientists as agents for environmental advocacy. Global Environmental Change.

[CR21] Jordan RC, Gray SA, Howe DV, Brooks WR, Ehrenfeld JG (2011). Knowledge gain and behavioral change in citizen-science programs. Conservation Biology.

[CR22] Kasperowski D, Hagen N (2022). Making particularity travel: Trust and citizen science data in Swedish environmental governance. Social Studies of Science.

[CR23] Kasperowski D, Hillman T (2018). The epistemic culture in an online citizen science project: Programs, antiprograms and epistemic subjects. Social Studies of Science.

[CR24] Kelling S, Fink D, LaSorte FA, Johnston A, Bruns NE, Hochachka WM (2015). Taking a ‘Big Data’ approach to data quality in a citizen science project. Ambio.

[CR25] Kitchin R (2014). Big Data, new epistemologies and paradigm shifts. Big Data and Society.

[CR26] Koepnick B, Flatten J, Husain T, Ford A, Silva DA, Bick MJ, Bauer A, Liu G (2019). De novo protein design by citizen scientists. Nature.

[CR27] Knape J, Coulson SJ, van der Wal R, Arlt D (2022). Temporal trends in opportunistic citizen science reports across multiple taxa. Ambio.

[CR28] Köttl H, Gallistl V, Rohner R, Ayalon L (2021). But at the age of 85? Forget it!: Internalized ageism, a barrier to technology use. Journal of Aging Studies.

[CR29] Kullenberg C, Kasperowski D (2016). What is citizen science? A scientometric meta-analysis. PLoS ONE.

[CR30] Lakomý M, Hlavová R, Machackova H, Bohlin G, Lindholm M, Bertero MG, Dettenhofer M (2020). The motivation for citizens’ involvement in life sciences research is predicted by age and gender. PLoS ONE.

[CR31] Land-Zandstra A, Agnello G, Gültekin YS, Vohland K, Land-Zandstra A, Ceccaroni L, Lemmens R, Perelló J, Ponti M, Samson R, Wagenknecht K (2021). Participants in citizen science. The science of citizen science.

[CR32] Larson LR, Cooper CB, Futch S, Singh D, Shipley NJ, Dale K, Takekawa JY (2020). The diverse motivations of citizen scientists: Does conservation emphasis grow as volunteer participation progresses?. Biological Conservation.

[CR33] Lemmens R, Antoniou V, Hummer P, Potsiou C, Vohland K, Land-Zandstra A, Ceccaroni L, Lemmens R, Perelló J, Ponti M, Samson R, Wagenknecht K (2021). Citizen science in the digital world of apps. The science of citizen science.

[CR34] Lewandowski E, Oberhauser KS (2017). Butterfly citizen scientists in the United States increase their engagement in conservation. Biological Conservation.

[CR35] Lewenstein BV (2022). Is citizen science a remedy for inequality?. The Annals of the American Academy of Political and Social Science.

[CR63] Lundquist E (2018). Flyktiga möten: Fågelskådning, epistemisk gemenskap och icke-mänsklig karisma.

[CR36] Mac Domhnaill C, Lyons S, Nolan A (2020). The citizens in citizen science: Demographic, socioeconomic, and health characteristics of biodiversity recorders in Ireland. Citizen Science: Theory and Practice.

[CR37] Mackenzie D, Wajcman J (1999). The social shaping of technology.

[CR38] Mannheim I, Schwartz E, Xi W, Buttigieg SC, McDonnell-Naughton M, Wouters EJ, Van Zaalen Y (2019). Inclusion of older adults in the research and design of digital technology. International Journal of Environmental Research and Public Health.

[CR39] McKinley DC, Miller-Rushing AJ, Ballard HL, Bonney R, Brown H, Cook-Patton SC, Evans DM, French RA (2017). Citizen science can improve conservation science, natural resource management, and environmental protection. Biological Conservation.

[CR40] Moczek N, Hecker S, Voigt-Heucke SL (2021). The known unknowns: What citizen science projects in Germany know about their volunteers - and what they don’t know. Sustainability.

[CR41] Mor Barak ME (2018). The practice and science of social good: Emerging paths to positive social impact. Research on Social Work Practice.

[CR42] National Academies of Science, Engineering and Medicine (2018). Learning through citizen science: Enhancing opportunities by design.

[CR43] Paleco C, García Peter S, Salas Seoane N, Kaufmann J, Argyri P, Vohland K, Land-Zandstra A, Ceccaroni L, Lemmens R, Perelló J, Ponti M, Samson R, Wagenknecht K (2021). Inclusiveness and diversity in citizen science. The science of citizen science.

[CR44] Pandya RE (2012). A framework for engaging diverse communities in citizen science in the US. Frontiers in Ecology and the Environment.

[CR45] Pateman R, Dyke A, West S (2021). The diversity of participants in environmental citizen science. Citizen Science: Theory and Practice.

[CR46] Peterson J, Kasperowski D, Van der Wal R, Travis C, Dixon DP, Bergmann L, Crampsie A (2022). Inter/national connections: linking nordic animals to biodiversity observation networks. Routledge handbook of the digital environmental humanities.

[CR47] Peterson, J., D. Kasperowski, and R. Van der Wal. 2023a. Bringing together species observations: a case story of Sweden’s biodiversity informatics infrastructures. *Minerva* 61: 265–289.

[CR64] Peterson, J. D., D. Kasperowski, and R. Van der Wal. 2023b. Does eBird contribute to environmental citizenship? A discursive analysis. *Environmental Communication *(in press).

[CR48] Phillips TB, Ballard HL, Lewenstein BW, Bonney R (2019). Engagement in science through citizen science: Moving beyond data collection. Science Education.

[CR49] Pocock MJO, Chandler M, Bonney R, Thornhill I, Albin A, August T, Bachman S, Brown PMJ (2018). A vision for global biodiversity monitoring with citizen science. Advances in Ecological Research.

[CR50] Purcell K, Garibay C, Dickinson JL, Dickinson JL, Louv R, Bonney R (2012). A gateway to science for all: celebrate urban birds. Citizen science: Public participation in environmental research.

[CR51] R Core Team (2020). R: A language and environment for statistical computing.

[CR52] Seymour V, Haklay M (2017). Exploring engagement characteristics and behaviours of environmental volunteers. Citizen Science: Theory and Practice.

[CR53] Sharma N, Greaves S, Siddharthan A, Anderson H, Robinson A, Colucci-Gray L, Wibowo AT, Bostock H (2019). From citizen science to citizen action: analysing the potential for a digital platform to cultivate attachments to nature. Journal of Science Communication.

[CR54] Silvertown J (2009). A new dawn for citizen science. Trends in Ecology and Evolution.

[CR55] Silvertown J, Harvey M, Greenwood R, Dodd M, Rosewell J, Rebelo T, Ansine J, McConway K (2015). Crowdsourcing the identification of organisms: A case-study of iSpot. ZooKeys.

[CR56] Spiers H, Swanson A, Fortson L, Simmons BD, Trouille L, Blickhan S, Lintott C (2019). Everyone counts? Design considerations in online citizen science. Journal of Science Communication.

[CR57] Sutherland WJ, Roy DB, Amano T (2015). An agenda for the future of biological recording for ecological monitoring and citizen science. Biological Journal of the Linnean Society.

[CR58] Theobald EJ, Ettinger AK, Burgess HK, DeBey LB, Schmidt NR, Froehlich HE, Wagner C, HilleRisLambers J (2015). Global change and local solutions: Tapping the unrealized potential of citizen science for biodiversity research. Biological Conservation.

[CR59] van Klink R, August T, Bas Y, Bodesheim P, Bonn A, Fossøy F, Høye TT, Jongejans E (2022). Emerging technologies revolutionise insect ecology and monitoring. Trends in Ecology and Evolution.

[CR60] Wickham H (2016). ggplot2: Elegant graphics for data analysis.

[CR61] Wood S (2006). Generalized additive models: An introduction with R.

[CR62] Wright DR, Underhill LG, Keenec M, Knight AT (2015). Understanding the motivations and satisfactions of volunteers to improve the effectiveness of citizen science programs. Society and Natural Resources.

