# Supplementary material for: Inequality persists in a large citizen science programme despite increased participation through ICT innovations
Source: Ambio. 2023 Sep 14;53(1):126–37. doi: 10.1007/s13280-023-01917-1 (PMC10692043; doi:10.1007/s13280-023-01917-1)
Supplement: Supplementary file 1 — Supplementary file1 (PDF 1433 KB) [file 13280_2023_1917_MOESM1_ESM.pdf]

Ambio

Electronic Supplementary material

Title: **Inequality persists in a large citizen science programme despite increased participation through ICT innovations**

Authors: Mari Jönsson, Dick Kasperowski, Stephen James Coulson, Johan Nilsson, Pavel Bína, Christopher Kullenberg, Niclas Hagen, René van der Wal, Jesse Peterson

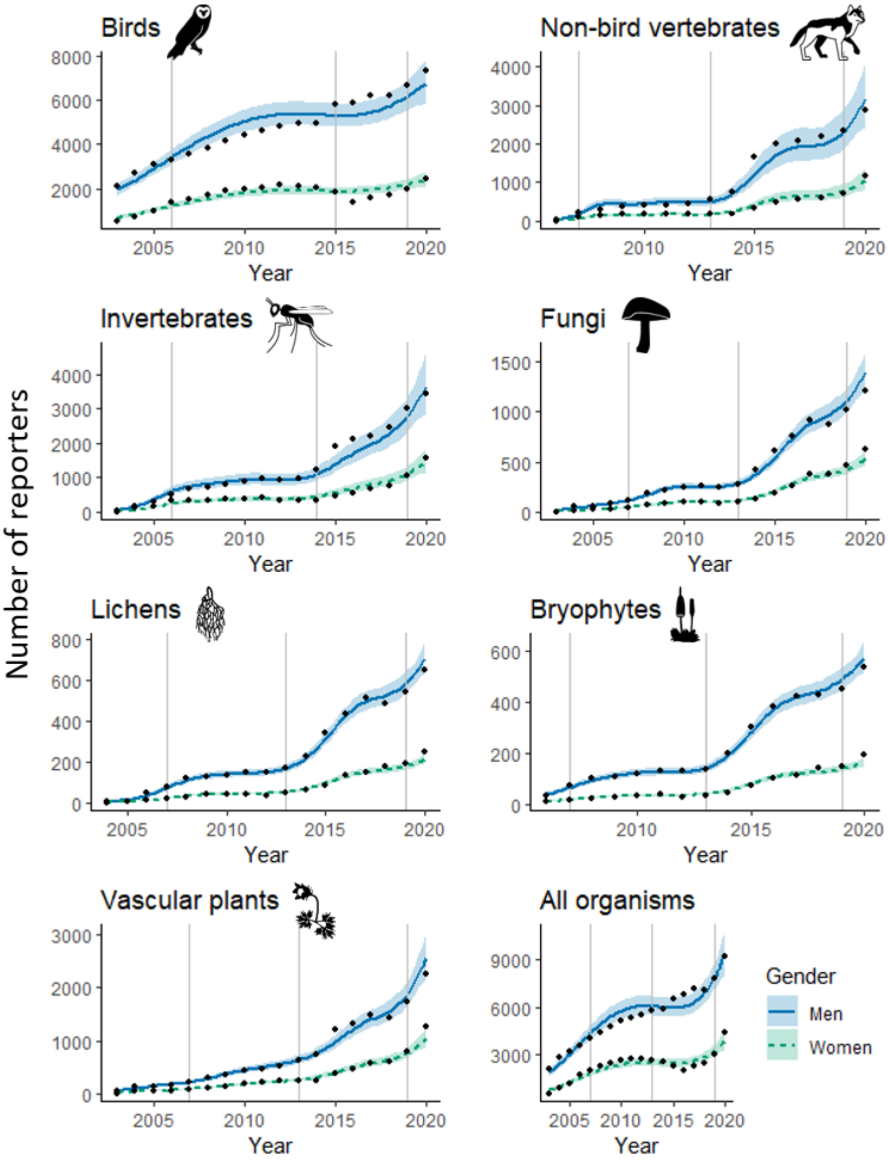

Figure S1. Estimated yearly number of men and women reporting for each species group (black dots represent raw data). Trends (with 95 % confidence intervals) are predicted values estimated from the generalized additive models. Vertical lines indicate times when new ICT developments were introduced (see Table 1 in main manuscript). Note that the scales of the y-axes differ.

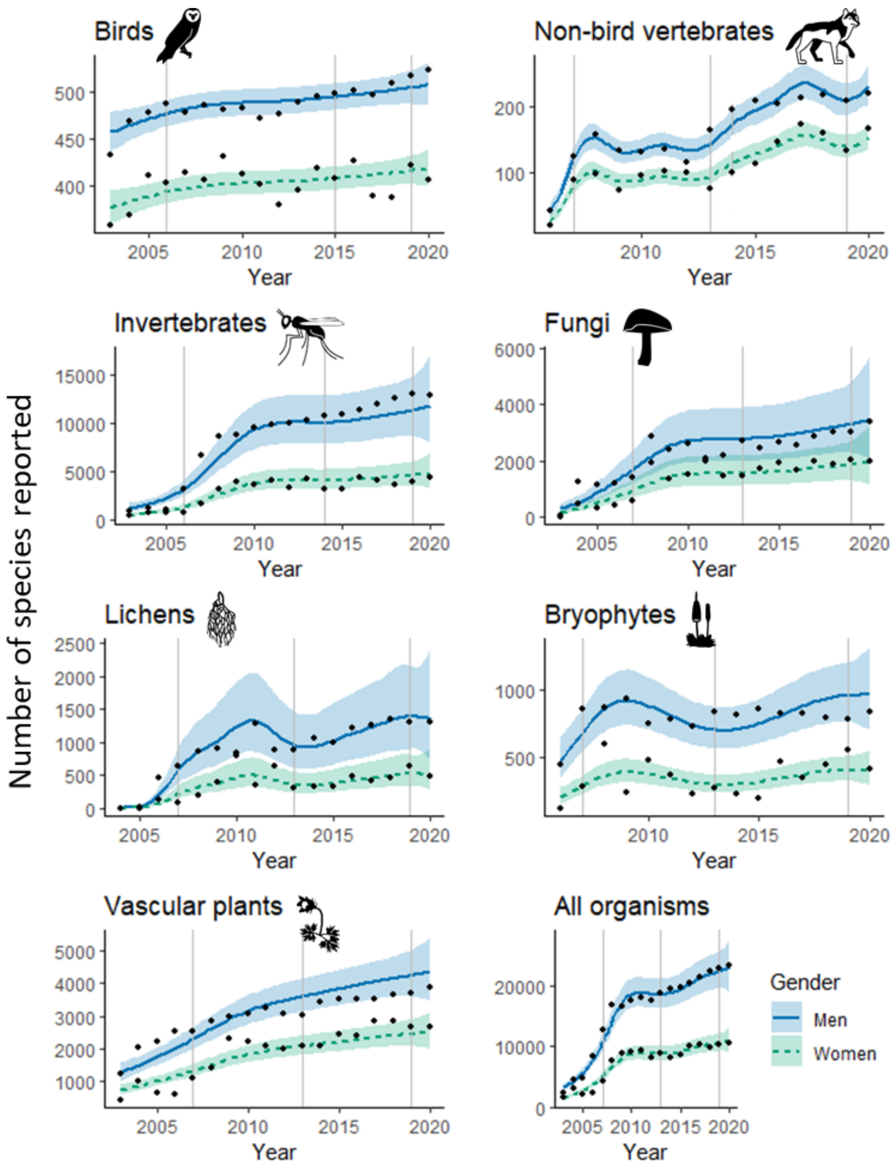

Figure S2. Estimated yearly number of unique species reported by men and women (black dots represent raw data). Trends (with 95 % confidence intervals) are predicted values estimated from the generalized additive models. Vertical lines indicate times when new ICT developments were introduced (Table 1 in main manuscript). Note that the scales of the y-axes differ.

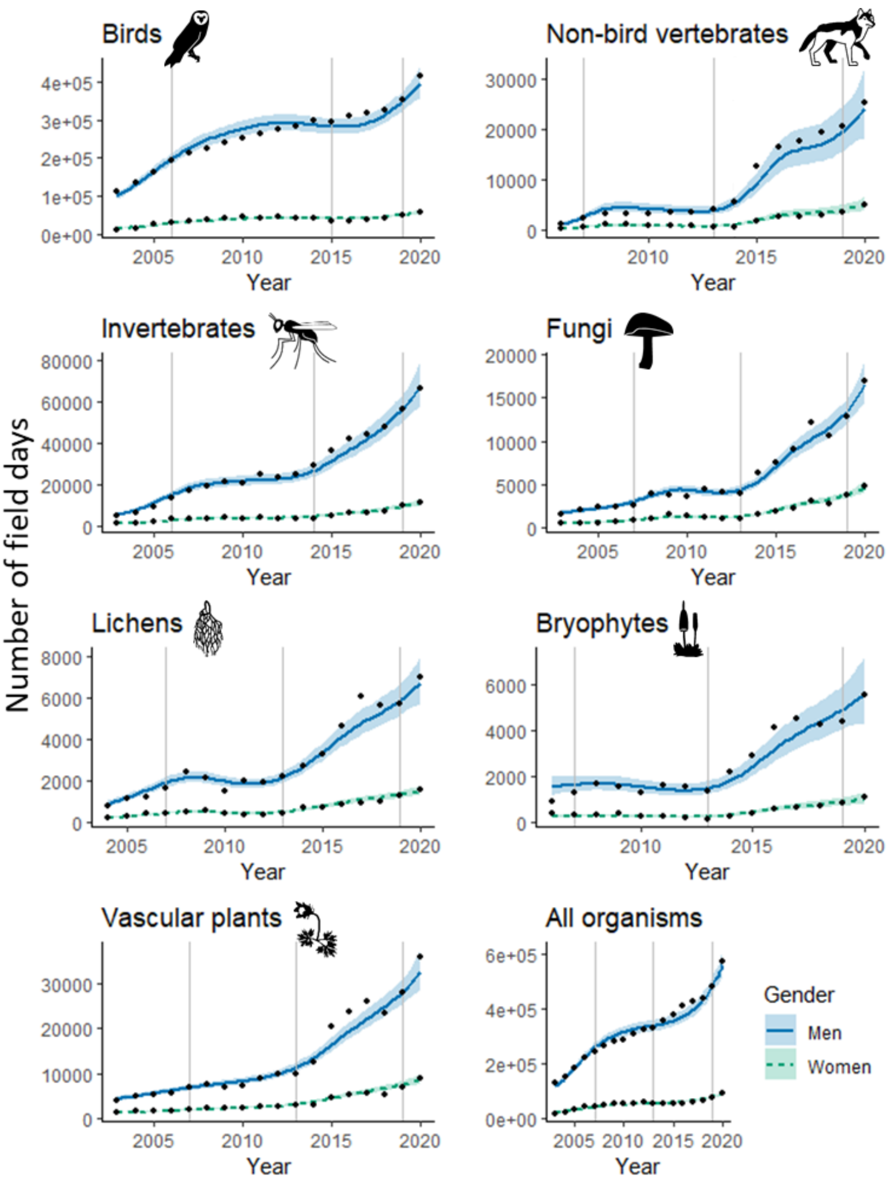

Figure S3. Estimated yearly number of days spent in the field by men and women (black dots represent raw data). Trends (with 95 % confidence intervals) are predicted values estimated from the generalized additive models. Vertical lines indicate times when new ICT developments were introduced (Table 1 in main manuscript). Note that the scales of the y-axes differ.

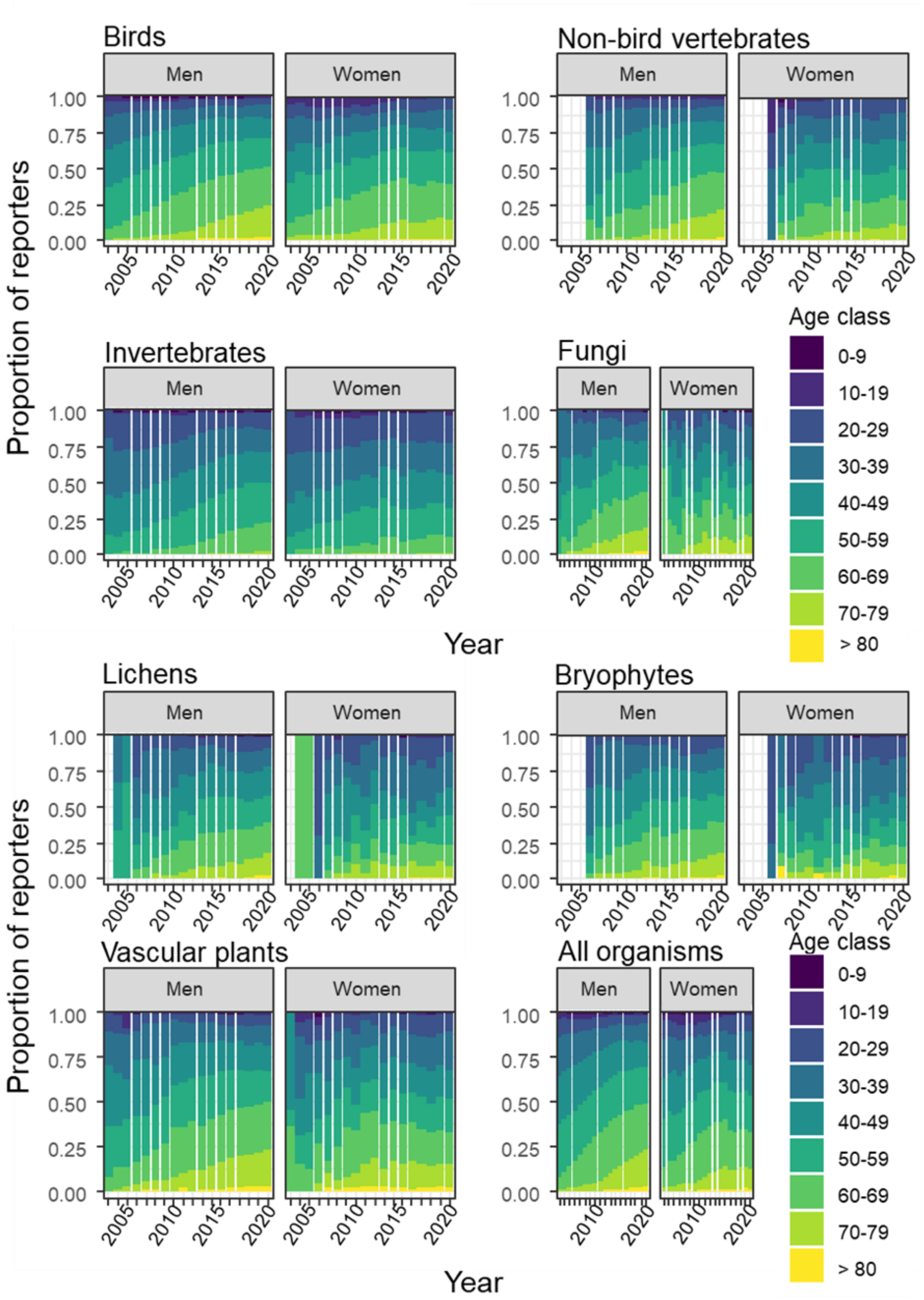

Figure S4. Age-class distribution of men and women reporting birds, non-bird vertebrates, invertebrates, fungi, lichens, bryophytes, vascular plants and all organisms to Artportalen 2003-2020.

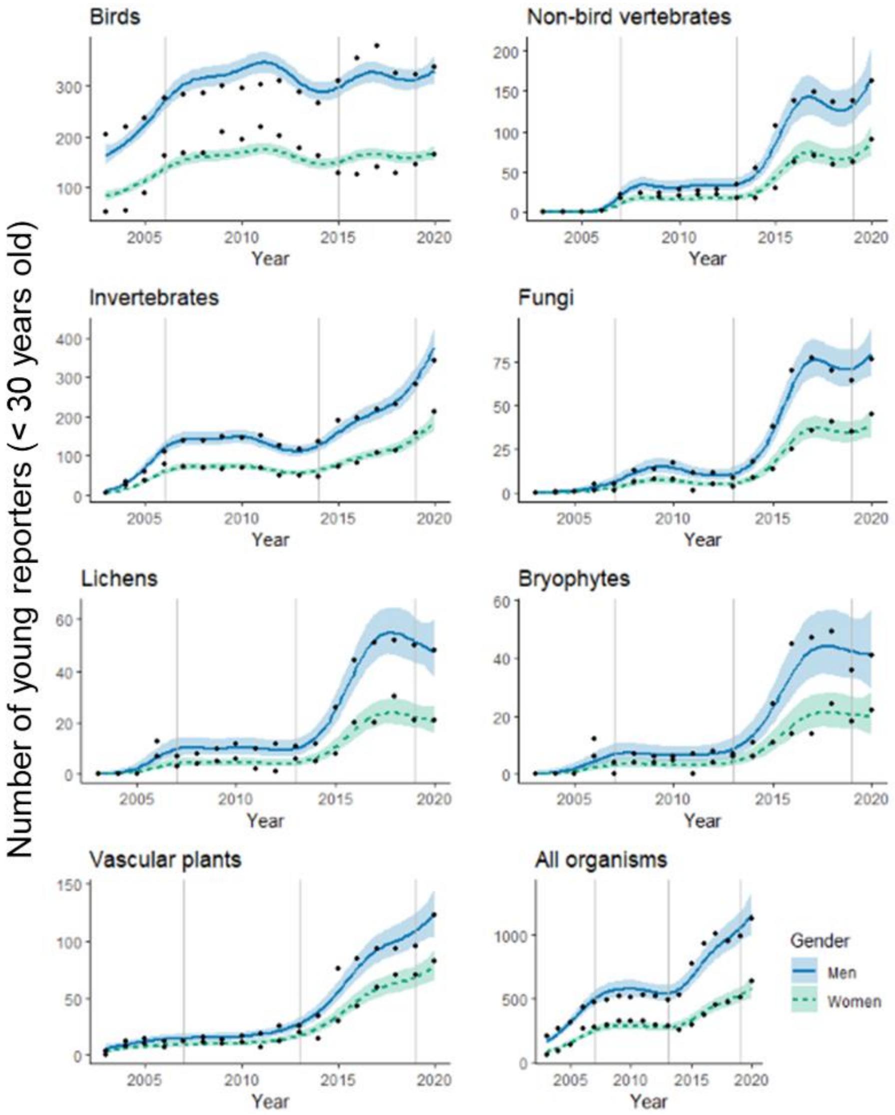

Figure S5. Estimated yearly number of young (< 30 years old) men and women reporting for each species group (black dots represent raw data). Trends (with 95 % confidence intervals) are predicted values estimated from the generalized additive models. Vertical lines indicate times when new ICT developments were introduced (see Table 1 in main manuscript). Note that the scales of the y-axes differ.

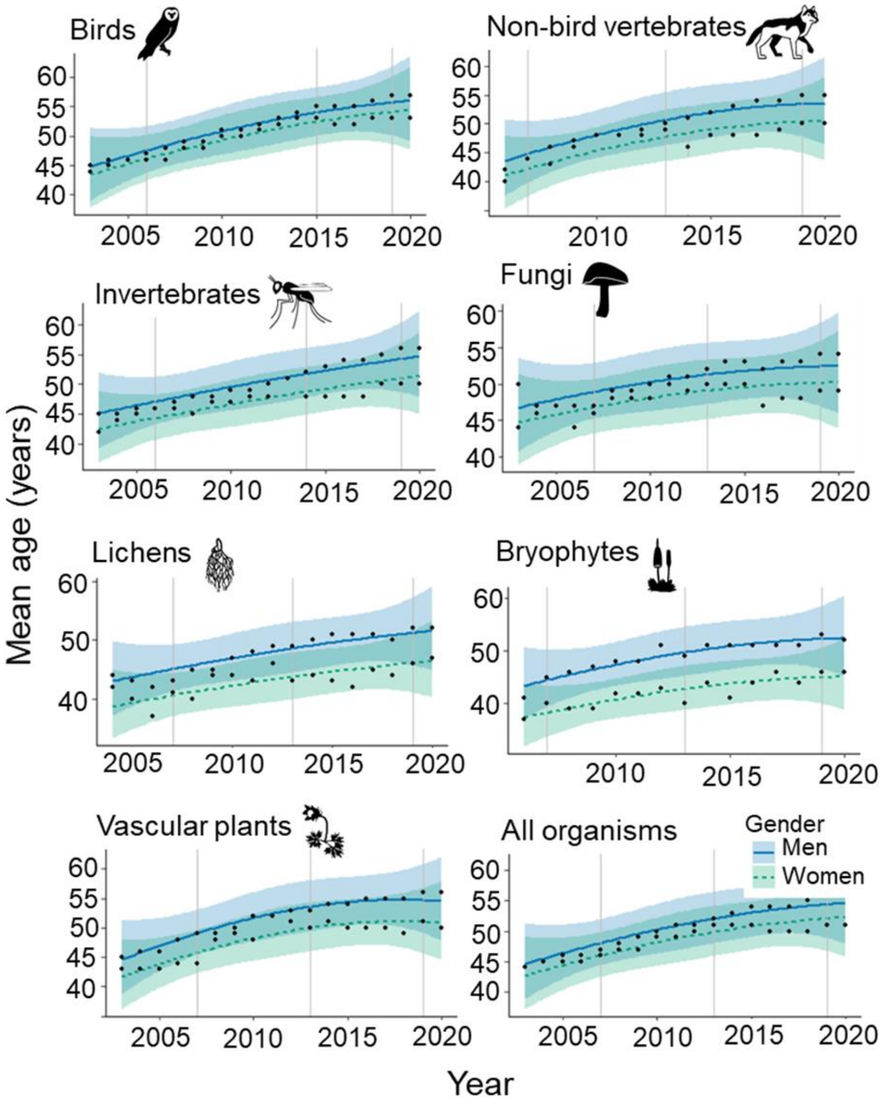

Figure S6. Estimated yearly mean reporter age of men and women (black dots represent raw data). Trends (with 95 % confidence intervals) are predicted values estimated from the generalized linear models. Vertical lines indicate times when new ICT developments were introduced (Table 1 in main manuscript). Note that the scales of the y-axes differ slightly.
